# Response to Letter to the Editor: “Conference on Medical Artificial Intelligence: Analysis Should be Based on Balanced Education and Audience”

**DOI:** 10.31662/jmaj.2025-0513

**Published:** 2025-12-12

**Authors:** Nafisa Islam, Abdel Rahman Osman

**Affiliations:** 1 Barking, Havering and Redbridge University Hospitals NHS Trust Queen’s Hospital, Romford, United Kingdom

**Keywords:** artificial intelligence, conference, healthcare

## To the Editors,

We thank Prof Shigeki Matsubara for their response to our study and are pleased that our paper was well received. Prof Matsubara ^(1)^ has raised many valuable points which we hope to address in the following response.

Addressing our sample size and selection first, we wholeheartedly agree that future work should seek to employ a larger sample size to increase statistical power and that conference attendees typically have fundamental artificial intelligence (AI) understanding as mentioned in our article. We would like to clarify that our paper states it is estimated that 100 attendees observed the National Health Service (NHS) National AI Conference at any given time as opposed to a single session ^(2)^.

With regards to the aims and design of the conference, we aimed to “educate health care professionals on defining AI, understanding its current uses and potential, the benefits and risks associated with integration in the NHS, and strategies to improve AI comprehension among the medical workforce”. To achieve this balanced approach, we involved multiple stakeholders, including four leading professionals in healthcare strategy and regulation, to ensure that we delivered a balanced and informed conference ^(2)^.

Finally, we would like to commend the suggestions Prof Matsubara ^(1)^ has put forward which will certainly guide future studies. For example, while our study was not designed to achieve this, comparing participant attitudes after teaching on AI safety as compared to AI benefits ^(2)^.

We are encouraged that our enthusiasm for educating medical professionals to make informed decisions about medical AI is shared.

## Article Information

### Author Contributions

We confirm that authors Nafisa Islam and Abdel Rahman Osman contributed equally to this work.

### Conflicts of Interest

None

### IRB Approval Code and Name of the Institution

None.
